# Effect of the Inclusion of *Bacillus* spp. in Growing–Finishing Pigs’ Diets: A Meta-Analysis

**DOI:** 10.3390/ani12172269

**Published:** 2022-09-01

**Authors:** Manuel Gonzalez-Ronquillo, Daniela Villegas-Estrada, Lizbeth E. Robles-Jimenez, Ricardo A Garcia Herrera, Vanessa L. Villegas-Vázquez, Einar Vargas-Bello-Pérez

**Affiliations:** 1 Facultad de Medicina Veterinaria y Zootecnia, Departamento de Nutrición Animal, Instituto Literario 100, Universidad Autónoma del Estado de México, Toluca 50000, Estado de Mexico, Mexico; 2 División Académica de Ciencias Agropecuarias, Universidad Juárez Autónoma de Tabasco, Carretera Villahermosa-Teapa, km 25, R/A La Huasteca 2a Sección, Villahermosa 86280, Tabasco, Mexico; 3 Department of Animal Sciences, School of Agriculture, Policy and Development, University of Reading, P.O. Box 237, Earley Gate, Reading RG6 6EU, UK

**Keywords:** probiotics, growth promoters, sustainable animal diet

## Abstract

**Simple Summary:**

Dietary probiotics are an alternative to antibiotic inclusion in pigs, the modulation of the intestinal environment, the inhibition of pathogen’s colonization by an increase in microbial competition in the gastrointestinal tract, and the regulation of mucosal immunity. These factors can lead to improvements in animal’s health and, therefore, productivity. The objective of this study was to use a meta-analysis approach to ascertain the effect of *Bacillus* spp. on growth performance of growing–finishing pigs and then to assess causes for the heterogeneity of responses detected using meta-regression. Overall, the inclusion of *Bacillus* spp. (median 486 mg/d) in growing–finishing pigs can increase the average daily gain (ADG) and decrease the feed: gain ratio (F:G).

**Abstract:**

This meta-analysis determined the effect of *Bacillus* spp. on growth performance of growing–finishing pigs and then assessed causes for the heterogeneity of responses detected using meta-regression. A database of 22 articles published from 2000 to 2020 was identified, and 9 articles fitted the selection criteria and were integrated in the final database. Statistical analysis was performed to analyze the effect size for ADG, average daily feed intake (ADFI), and F:G ratio using a standardized means difference (SMD) at a 95% confidence interval. A meta-regression analysis was used to investigate the cause of heterogeneity, using the individual SMD for each study assessment as the outcome and the associated SE as the measure of variance. Dietary *Bacillus* spp. supplementation had no effect on ADFI (SMD: −0.052, *p* = 0.138) and numerically increased ADG (SMD: 0.113, *p* = 0.081) and reduced the F:G ratio SMD: −0.127, *p* < 0.001). Meta-regression outcomes suggested that the number of animals per group was an essential component promoting heterogeneity in ADG. Overall, the inclusion of *Bacillus* spp. (median 486 mg/d) in growing–finishing pigs can increase ADG and can decrease the F:G ratio.

## 1. Introduction

Antibiotic resistance is a global topic of great concern among the scientific community. By 2010, the antimicrobial consumption in the livestock sector was 63,151 tones worldwide [1,2,3]. Antibiotics have been used in swine production in sub-therapeutic dosages as growth promoters for increasing production efficiency [4], feed utilization efficiency [4], and reproductive performance [5]. The inclusion of antibiotics in diets has been routinely used to prevent or treat diseases by reducing mortality and morbidity [6], with the risk of antimicrobial resistance.

Antibiotics were first approved in 1951 by the Food and Drug Administration (FDA) [7,8] as feed additives for farm animals. Antibiotics were demonstrated to be so effective that the swine population reached 1 million by 2015, which was double the population in the 1970s [9,10]. On the other hand, the misuse of antibiotics can cause antibiotic resistance, and this has led to a reduction in their utilization. Several countries, such as Korea 2011 [3,11] and those in the European Union 2016 [12,13], confirmed their entry to the “Post-antibiotic Era” by completely banning antibiotic usage as growth promoters.

Today, many countries have been entering the “Post antibiotic era”, which is reflected in the inclusion of probiotics in pigs’ diets as a substitute for antibiotics usage [14]. Dietary probiotics provide a potential alternative to reduce antibiotic inclusion in pigs with the following benefits: the regulation of the intestinal environment [15], the inhibition of pathogen’s colonization by an increase in microbial competition in the gastrointestinal tract [16], and the regulation of mucosal immunity [15]. All these factors can lead to improved animal’s health and, therefore improved weight gain, feed intake, feed conversion ratio, dry matter digestibility [17], and nitrogen digestibility [18]. In this regard, the use of different Bacillus strains has led to inconsistent and contradictory results, which is why the utilization of these probiotics is still being questioned [19]. Alexopoulos et al. [20] mentioned that the dietary supplementation of *Bacillus subtilis* and *Bacillus licheniformis* improved the average daily gain (ADG) and average daily feed intake (ADFI) compared to the control group in pigs. On the other hand, Giang et al. [21] reported no effects on pigs’ growth performance for *Bacillus subtilis* supplemented in growing and finishing pig’s diets. Currently, little research has been performed to clarify if these inconsistencies in the use of Bacillus probiotics depend on characteristics of the strains, the amount used, the type of animal breed used, or whether it is influenced by the growth period in which it is used (*Bacillus coagulans*-*fermented*, [22]; *Bacillus coagulans* GBI-30 and 6086, [23]).

A meta-analysis is a statistical tool that relates results from several investigations and composes them statistically [24]. This meta-analysis determined the effect of *Bacillus* spp. on the growth performance of growing–finishing pigs and analyzed causes for the heterogeneity of responses observed using meta-regression.

## 2. Materials and Methods

### 2.1. Search for Studies

A thorough exploration of available studies in English was conducted from 1990 to 2020 to identify experiments centered on analyzing the impact of *Bacillus* spp. on growing–finishing pigs’ production performance. The search included the following databases: ISI Web of Knowledge (http://wokinfo.com, accessed on 1 May 2021) ), Google Scholar (http://scholar.google.com, accessed on 10 May 2021), Scopus (https://www.scopus.com, accessed on 30 May 2021), Science direct (https://www.sciencedirect.com, accessed on 30 May 2021), BMC Research (https://bmcresnotes.biomedcentral.com accessed on 15 May 2021), PubMed (https://pubmed.ncbi.nlm.nih.gov, accessed on 29 May 2021), and Redalyc (https://www.redalyc.org, accessed on 23 May 2021). The keywords used included Bacillus, pig, and performance, and results were arranged in an order of importance. The assessment of documents ended when 50 sequential citations were not relevant. The reference lists of all articles compiled were verified to ensure that no pertinent studies were ignored. Only data from full texts from electronic databases, from interlibrary loans, or by contacting the authors were used for the meta-analysis [25].

### 2.2. Inclusion and Exclusion Criteria

**Figure 1:**
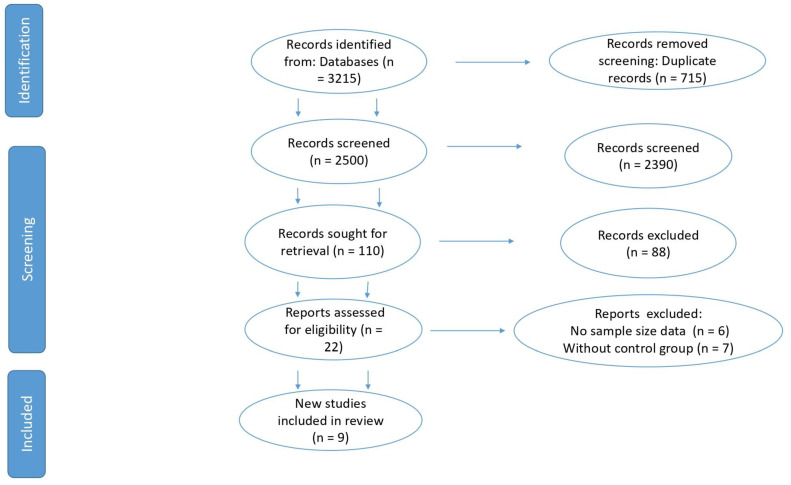
PRISMA flow diagram of the systematic review from the initial search and screening to the final selection of publications included in the study.

This study followed PRISMA guideline and Figure 1 shows a flow diagram [26] of the collected data. After preliminary search and screening, 22 articles were evaluated to be eligible. Seven were excluded due to the following reasons: Failure to provide the required statistical parameters (*n* = 3) and not having a control group (*n* = 4). Therefore, nine articles were found for this meta-analysis as they had the primary inclusion criteria. Two assessors assessed all articles using the inclusion and exclusion criteria. A listing of studies contained in the meta-analysis is shown in Table 1.

### 2.3. Data Mining

Data obtained from each study considered authors’ names,; the year of publication; initial body weight (kg); experiment period in days; average daily gain (kg/d); average daily feed intake (kg/d); F:G; the number of animals in experimental groups (control and treatments); and standard error (SE). The standard deviation (SD) was noted as the measure of variance. If SD was not reported in studies, it was calculated by multiplying the reported SE of means by the square root of the sample size. Selected articles did not report dietary energy and protein contents in most cases, and this prevented the use of these parameters as covariates for meta-regression.

Data were transferred to Excel spreadsheets (version 2016, Microsoft Corp., Redmond, WA, USA). The search was performed by two authors to avoid reviewer bias and to assure that compiled data were correctly recorded from the documents into the spreadsheets before statistical analyses.

### 2.4. Statistical Analysis

#### 2.4.1. Effect size and Forest Plots

Statistical analysis was performed using Comprehensive Meta-Analysis (CMA) software version 3 (Biostat, Florida, United States of America) to calculate the effect size for the average daily feed intake, average daily gain, and F:G in terms of standardized means difference (SMD) at a 95% confidence interval. The SMD indicates the mean difference between treatment and control groups standardized based on SD of treatment and control groups [35]. The SMD was calculated. by applying the following formula:

$$SMD=\frac{{\bar{x}}_{t}-{\bar{x}}_{c}\;}{S_{p}}$$

where ${\bar{x}}_{t}$ is the treatment group mean, ${\bar{x}}_{c}$ is the control group mean, and $S_{p}$ is the pooled SD [36].

In addition, for SMD calculations for each result, the raw mean difference (RMD) was calculated with a 95% confidence interval. A random-effect model was used for the meta-analysis, which has a fundamental assumption that the distribution of effects exists, resulting in heterogeneity among the studied results [35]. The significance of the effect size estimates was declared at *p* ≤ 0.05. Forest plots were constructed to evaluate the effect of *Bacillus* spp. on the average daily feed intake (kg/d), average daily gain (kg/d), and F:G. The effect size for forest plots was the SMD at the 95% confidence interval using the random-effect model.

#### 2.4.2. Heterogeneity

Statistical heterogeneity indicates that the true effects in each study were identical [24], and it reflects inherent differences in the diversity of pig herds, differences in study design, and statistical variations [36]. Chi-square (Q) tests and the I^2^ statistic were used to measure heterogeneity [35]. Variations among the study level were assessed using a Q test, and the significance level was set at 0.1 [36,37]. Although the Q test is helpful in identifying heterogeneity, the measure of I^2^ was used to measure heterogeneity as a percentage [36]. Negative values of I^2^ were equal to zero; consequently, I^2^ lies between 0 and 100% [36].

#### 2.4.3. Meta-Regression

Meta-regression was used to investigate the cause of the heterogeneity of responses, applying the individual SMD for each study comparison as the result and the related SE as the measure of variance. Meta-regression was performed following the DerSimonian and Laird method [35]. In this study, the variables of initial body weight, experiment period, and the number of animals in experimental groups were used as covariates for data related to heterogeneity.

#### 2.4.4. Publication Bias

Egger’s linear regression asymmetry was used to investigate the existence of publication bias [38]. When significant (*p* < 0.10) bias was detected, the trim-and-fill method [39] was applied to find the number of missing observations.

## 3. Results

Table 1 indicates the main attributes of each of the selected studies. In general, the studies were conducted in a homogeneous number by sex 50/50 for females and males, except for Balasubramanian et al. [27], Giang et al. [21], and Upadhaya et al. [28] who used a ratio of two gilts and three barrows per treatment. The main breeds in the present meta-analysis were [Yorkshire × Landrace] × Duroc, except for Giang et al. [21], who used Yorkshire × Landrace, and Nitikanchana et al. [29], who used PIC 1050 × 337 [(Large White × Landrace) × (Pietrain × Duroc)]. The average initial live weight ranged from 23.3 to 60.0 kg, and the mean duration of the experiments was 88 ± 12 days. The present studies used several species of *Bacillus* spp., including *B. subtillis*, *B. licheniformis*, *B. coagulans*, and *B. amyloliquefaciens*. Giang et al. [21], Patarapreecha et al. [30], and Silva et al. [31] used only *B. subtilis*, and Fu et al. [32] used *B. coagulans*, while Rybarczk et al. [33] and Upadhaya et al. [28] used a combination of *B. licheniformis* and *B. subtilis* and van der Peet-Schwering et al. [34] used a combination of *B. subtilis* and *B. amyloliquefaciens*. Balasubramanian et al. [27] was the only one who used a combination of three bacillus, *B. licheniformis*, *B. subtilis*, and *B. coagulans.* Finally, Nitikanchana et al. [29] used trademark Sporzyme^®^, which does not indicate the species used. *Bacillus* spp. doses ranged at 1048.6 ± 1796.0 mg/d, with a minimum of 48.6 mg/d [40] and maximum of 6480 mg/d [21]; the median ranged at 469.9 mg/d. The ME (MJ/kg DM) in the diets was between 13 and 14 MJ ME/kg DM, except for Rybarczk et al. [33], which was lower (11.70 MJ ME/kg DM). Crude protein (CP) concentrations ranged from 138 to 185 g/kg DM, apart from Fu et al. [32] and Patarapreecha et al. [30] (126 and 144 g/kg DM, respectively). Dietary Lys concentrations in general were between 9.1 and 9.8 g/kg DM, except for Fu et al. [32], Patarapreecha et al. [30], and Silva et al. [31], which were lower.

**Figure 2:**
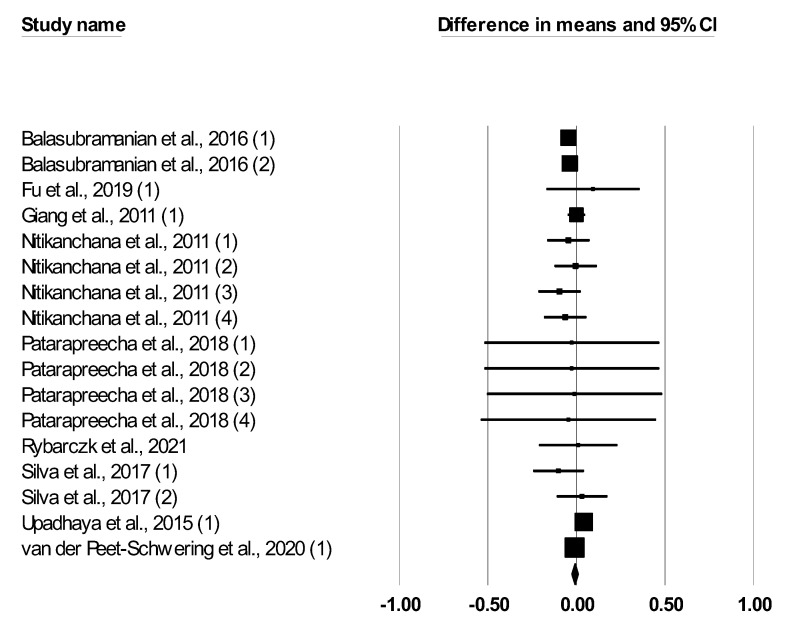
Forest plot of the effect of *Bacillus* spp. on mean daily feed intake in growing and finishing pigs, based on standardized mean differences (Std. diff in means). The diamond at the bottom indicates the mean effect size, while the size of the squares illustrates the weight of each study in relation to the mean effect size. Smaller squares represent smaller weights. The confidence intervals (95% for the study) are represented by horizontal bars.

**Figure 3:**
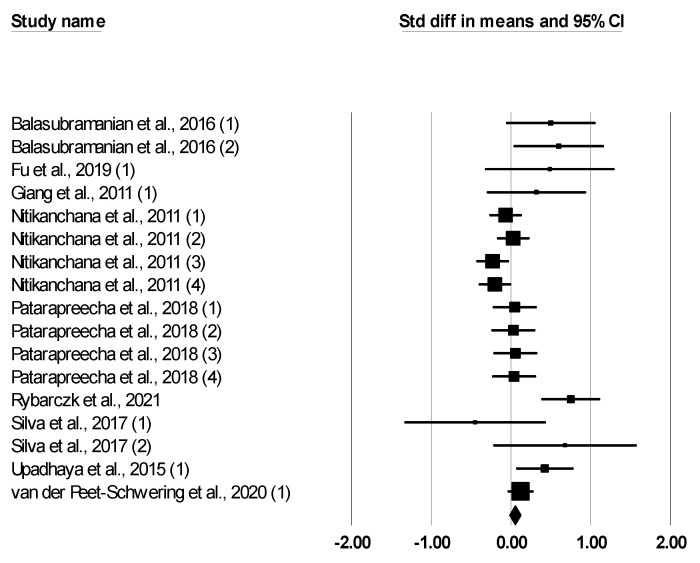
Forest plot of the effect of *Bacillus* spp. on average daily gain in growing–finishing pigs based on standardized mean differences (Std. diff in means). The diamond at the bottom indicates the mean effect size, the size of the squares illustrates the weight of each study in relation to the mean effect size, and horizontal bars represent confidence intervals (95% for the study).

**Figure 4:**
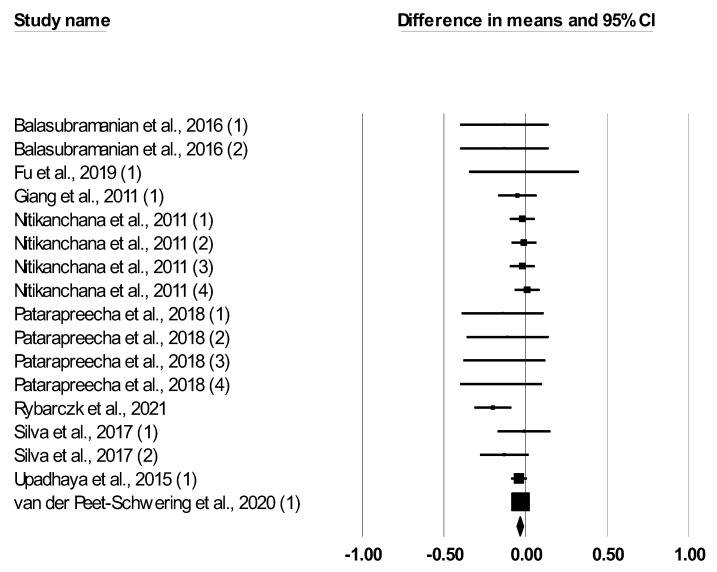
Forest plot of the effect of *Bacillus* spp. on feed:gain ratio in growing–finishing pigs based on standardized mean differences (Std. diff in means). The size of the squares illustrates the weight of each study, smaller squares represent lower weight, and the diamond indicates the mean effect size. Horizontal bars represent confidence intervals (95% for the study).

The meta-analysis findings for the effect of *Bacillus* spp. on the performance of growing–finishing pigs are reported in Table 2. Dietary *Bacillus* spp. supplementation had no effect on ADFI (SMD: −0.052, *p* = 0.138). Dietary *Bacillus* spp. supplementation tended to increase ADG (SMD: 0.113, *p* = 0.081) and decreased the F:G ratio (SMD: −0.127, <0.001). The forest plot (Figure 2, Figure 3 and Figure 4) showed outcomes of individual studies and the overall outcome for ADFI, ADG, and F:G ratio. Heterogeneity was not significant for ADFI and F:G, but it was significant for ADG (Q and I2, Table 2).

Meta-regression results indicated that the number of animals per group was an important factor promoting heterogeneity in ADG (Table 3). No publication bias was observed for ADFI (*p* > 0.10, Table 2). Publication bias was detected for the ADG and F:G ratio (*p* < 0.10). The trim-and-fill method indicated five missing observations for the average daily gain and F:G ratio.

## 4. Discussion

The present meta-analysis used nine studies on growing–finishing pigs to ascertain the consequences of *Bacillus* spp. on their performance. Differences in the amounts used (mg/d) were observed (Table 1), and these inconsistencies may be primarily due to the range of action on complex and variable feed ingredients [40]. 

Regarding breeds and crossed animals used in the experiments (Table 1), Zimmermann et al. [41] analyzes the probiotic effect on ADG and crossbreeding F1 pigs. When crossbreeds, pure breeds, and backcrossing were used in experiments, the probiotic effect on ADG could not be detected. However, in the present study, we observed a trend (*p* = 0.08) on ADG, and these results coincide with Zimmerman et al. [41], who reported that probiotics modify ADG when there were included in experiments using maternal breeds (e.g., Landrace and Yorkshire) and the crossbreeding of prolific and rapid growth breeds (e.g., Berkshire and Duroc). Therefore, we can infer that the use of F1 crossbreeding expresses hybrid vigor with the addition of *Bacillus* spp. in the diet compared to pure breeds [41]. In our search for scientific reports, we were unable to find studies related to the influence of genetic selection and crossbreeding with the supplementation of probiotics. This is important as rates of weight gain can be influenced by genetic types, diet, sex, genetic type and diet interactions, and diet and sex interactions [42].

The inclusion of *Bacillus* spp. in the present study did not generate differences (*p* >0.05) in ADFI (Table 2). This result coincides with Alexopoulos et al. [20], who used *B. lincheformis* and *B. subtillis* in growing–finishing pigs (ADFI; *p* > 0.05) and Giang et al. [21] who used *Bacillus subtillis* in finishing pigs (ADFI; *p* = 0.095). Quite the opposite, Lee et al. [43] showed that the addition of the Bacillus complex (0.15, 0.3 and 0.45 mg/kg) in the diet improved ADFI (*p* > 0.01) when Bacillus concentrations were improved in the piglet diet compared to the control group and increased ADG. Clear results on growth performance were observed in the early period after weaning by adding the lactobacilli complex, but there were no positive effects in later periods after weaning [44]. Regarding growing and finishing pigs, the administration of probiotics in high-energy and high protein density diets is more effective than in low-density diets [16,45]. However, all diets of the present study fulfilled nutritional requirements according to the NRC [46], without observing an effect on the increase in the body mass index. However, a trend in the body mass index was observed, which resulted in a greater efficiency in the utilization of nutrients ingested by a pig when supplemented with Bacillus. In addition, our results suggest that improved nutrient utilizations occurred, and this was reflected in a lower F:G ratio, which led to accelerated metabolisms and the transformation of feed into body mass (ADG and FCR) and the transformation into lean meat [47]. This could be because Deng et al. [48] reported that adding Bacillus in piglet diets enhances the action of lipids, proteins, and carbohydrate metabolisms and promotes mucosal maturity in piglets, in addition to increasing the height of the ileal villus and depth of the duodenal crypt [49].

The ADG and F:G ratio values from this meta-analysis coincided with Jonsson and Conway [50] and Davis et al. [51], who explained that the dietary supplementation of *Bacillus* spp. increases the growth performance of pigs. This effect was not observed with the inclusion of other probiotics, which was demonstrated in a meta-analysis conducted in three studies, where Zimmerman et al. [41] mentioned that the use of *Enterococcus* spp., *Pediococcus* spp., *Lactobacillus* spp., and *Saccharomyces* spp. did not improve performances in pigs. The dietary supplementation of *Bacillus* spp. as feed additives improved the performance of the ADG and F:G ratio, which agrees with prior observations [52] in weaning pigs. 

The results of a meta-analysis performed by Zimmerman et al. [41] indicated that adding probiotics improved the F:G ratio at all stages of production. This increase in ADG with a lower F:G ratio may be because, during post-weaning, the digestive tract of piglets is still not completely developed, and the capacity to digest nutrients is at a minimum. It is known that some digestive enzymes generated by Bacillus could promote the absorption of nutrients and improve animal’s feed conversion [49]. Earlier findings also indicated that dietary supplementation with *B. subtilis* can boost the quantity of Lactobacilli in the gastrointestinal tract of pigs, thus decreasing the proliferation of *Escherichia coli* and stimulating microbial flora balance [53,54]. Therefore, supplementation with *B. subtilis* may have a positive impact on the growth performance of weaned piglets, which is possibly due to enhanced endocrine hormone concentrations and enhanced health status [52].

The inclusion of *Bacillus* spp. in the diet increased the ADG (Table 2) when compared to the control group (Table 2). Similar results for ADG and the increase in growth performance including *Bacillus* spp. treatments were reported by several authors [21,27,28], increasing from 3 to 5%. The present results agree with Zimmermann et al. [41], where probiotic supplementations had beneficial effects on ADG (29.939 g) and feed efficiencies (96 g less feed consumed/kg of weight gain).

The dietary inclusion of microbial feed additives, as growth promoters, generally showed an enhancement in growth performance and digestibility, as there is a benefit to the host microflora through microbial balance in the gut [55]. Furthermore, Davis et al. [51] reported that *Bacillus* spp. increased the expected mean for ADG in growing–finishing pigs supplemented at 0.5% and provided 1.47 × 10^8^ CFU/g of the supplement with *Bacillus* spp. Balasubramanian et al. [56] had a linear trend (*p* = 0.052) on ADG at week 16 (an increase of 5%) and a significant linear effect on ADG (*p* = 0.041) (an increase of 3%) during the overall experiment when supplementing growing–finishing pig diets with *Bacillus* spp.

*Bacillus* spp. are used as additives because they are stable as spore-forming bacteria that resist heat, enzymatic degradation, and acidic conditions of the stomach [57]. They also produce a variety of enzymes such as amylases, cellulases, lipases, and proteases, arabinase, dextranase, levansucrase, maltase, alkaline protease, and β-glucanase [17,58] that facilitate excretion and digestion [59]. Moreover, *Bacillus* spp. enhance the pig’s immune system; they can regulate the symbiotic microbiota of the host and inhibit the growth of pathogenic microorganisms [57], since some antibiotics are extracted from them as is the case of *B. Linchenformis*, which is used to produce bacitracin [60]. In this sense *B. amyloliquefaciens* produces different lipopeptides and four polypeptide compounds, which are known antimicrobials [61].

*B. subtilis* consumes oxygen in the intestinal tract and produces enzymes such as subtilisin and catalase. This results in a positive environment for lactobacilli (which produce lactic acid), which colonize the mucous membranes of the intestine and block adhesion sites for pathogens, such as Clostridium, *E. coli*, Salmonella, and Campylobacter, with a reduced incidence of diarrhea [57,62] and improved growth performance [63] in piglets.

Chesson [64] reported that constant inoculation is necessary to keep probiotic organisms in the digestive tract, as bacterial counts in the intestine return to pre-feeding levels approximately 24 h later. 

The meta-regression results indicated that the number of animals per group influences ADG heterogeneity. The number of animals per group is from 10 to 288 between studies (Table 1). This suggests that with an increasing number of animals per group, the ADGs decrease. These findings should be a focus of attention since they were generated from a few studies.

## 5. Conclusions

The effect dietary inclusion *Bacilllus* spp. in growing–finishing pigs had no effect on the average daily feed intake and tended to increase the average daily gain. Overall, the inclusion of *Bacillus* spp. (median 486 mg/d) in growing–finishing pigs can increase ADG levels and can decrease the F:G ratio. Meta-regression results indicated that the number of animals per group was a significant component that contributed to heterogeneity in the average daily feed intake.

## Figures and Tables

**Table 1:** Papers used for meta-analysis in growing finishing pigs supplemented in pig diets with *Bacillus* spp.

Reference	^1^ NC	Breed	Sex	Based Diet	^2^ IBW	Td (days)	^3^ N	^4^ CP g/kg	^5^ ME MJ/kg	^6^ Anti	*Bacillus* spp.	*Bacillus* spp. (mg/d)	^7^ Ca	^8^ P	^9^ Lys	^10^ Met
Giang et al. [21]	1	[Yorkshire × Landrace]	8 gilts and 12 barrows per treatment	Corn-SBM	28.70 ± 0.90	42	20	19.4 ± 1.1	14.45	NU	*B. subtilis* H4 (6 × 10^11^ CFU/mL)	6480.0	9.1	4.0	9.0	2.9
Balasubramanian et al. [27]	2	[(Yorkshire × Landrace) × Duroc]	Three barrows and two gilts per pen	SBM	23.3 ± 1.40	112	25	185.6 ± 15	13.21	NU	*B. coagulans* (1 × 10^9^ cfu/g), *B. lichenformis* (5 × 10^8^ cfu/g), *B. subtili*s (1 × 10^9^ cfu/g)	211.7–426.8	8.0	5.1	9.8	2.9
Upadhaya et al. [28]	1	[(Yorkshire × Landrace) × Duroc]	2 gilts and 3 barrows per pen	Corn-SBM-Wheat	23.6 ± 1.41	112	60	182.8 ± 11	NR	UN	*B. Linchenformis* and *B. subtilis* (1.47 × 10 ^8^ CFU/g)	NR	8.0	5.0	9.5	2.8
Nitikanchana et al. [29]	4	PIC 1050 × 337 [(Large White × Landrace) × (Pietrain × Duroc)]	NR	Corn-SBM	34.01	105	183	180 ± 15	14.08	NU	Sporzyme ^®^ (4.36 × 10^12^ CFU/lb)	NR	5.0	4.5	9.3	0.05
Patarapreecha et al. [30]	4	[(Yorkshire × Landrace) × Duroc]	Half barrows and half gilts	Corn-SBM	60 ± 1.2	52	100	180.1 ± 10	12.57	NS	*B. subtilis* (1.0 × 10^12^ CFU g)	248.0–1989.0	2.28	ND	2.2	0.2
Silva et al. [31]	2	[Large White × Landrace]	Half barrows and half gilts	Corn-SBM	26.07 ± 0.07	82	10	171.0 ± 15	13.73	Lincomycin	*B. subtilis* C-3102 (1.0 × 10^10^ CFU g)	66.0–78.9	6.5	2.9	7.5	2.4
Fu et al. [32]	1	[(Yorkshire × Landrace) × Duroc]	NR	Corn-SBM	26.87 ± 2.65	105	12	138.8 ± 11	14.23	Enramycin	*B coagulans* (5.0 × 10^9^ cfu/g)	48.6	4.6	3.9	6.1	1.8
Rybarczyk et al. [33]	1	[(Yorkshire × Landrace) × Duroc]	Half barrows and half gilts	Wheat -Triticale	33.15 ± 2.36	77	60	166.5 ± 15	11.70	NU	*B. licheniformis* DSM 5749 (1.6 × 10^9^ CFU/g), and *B. subtilis* DSM 5750— (1.6 × 10^9^ CFU/g)	1012.0	5.8	4.5	9.8	2.8
van der Peet-Schwering et al. [34]	1	[(York × Dutch Landrace) × Large White boar]	6 barrows and 6 gilts per pen	Corn-SBM-Wheat-Barley	23.2 ± 2.95	102	288	174.5 ± 1	14.33	NU	*B. amyloliquefaciens* DSM 25,840 and *B. subtilis* DSM 3232 (1.5 × 10^9^ CFU/g)	784.0	4.7	ND	ND	ND

^1^ NC: Number of comparisons; ^2^ IBW: initial body weight; Td (days): treatment duration (days); ^3^ N Anim: number of animals per treatment; *Bacillus* spp. doses (mg/d); ^4^ CP, crude protein; ^5^ ME, metabolizable Energy (MJ/kg DM);^6^ Anti: control group include dietary antibiotics; ^7^ Ca, calcium (g/kg DM); ^8^ P, phosphorous (g/kg DM); ^9^ Lys, lysine (g/kg DM);^10^ Met, methionine (g/kg DM); ND, not reported; NU, non-use of antibiotics; SBM, soybean meal; Sporzyme ^®,^ (Micro Source S, DSM Nutritional Products, Basel, Switzerland).

**Table 2:** Effect size and heterogeneity for the effect of *Bacillus* spp. on the growth performance of growing–finishing pigs.

		SMD ^2^ (95% CI ^3^)		Heterogeneity			RMD ^4^ (95% CI)	Publication Bias
Outcomes ^1^	No. of Comparisons	Random Effect	*p-Value*	Q	*p-Value*	I ^2^	Random Effect	Egger
ADFI, kg/d	17	−0.052 (−0.120, 0.017)	0.138	15.123	0.516	0.00	−0.012 (−0.029, 0.006)	0.829
ADG, kg/d	17	0.113 (−0.014, 0.240)	0.081	43.704	<0.001	63.39	0.011 (−0.003, 0.025)	0.033
F:G ratio	17	−0.127 (−0.195, 0.058)	<0.001	15.048	0.521	0.00	−0.037 (−0.056, −0.017)	0.060

^1^ ADFI: average daily feed intake; ADG: average daily gain; F:G: feed: gain ratio. ^2^ SMD: standardised mean difference. ^3^ CI: confidence interval. ^4^ RMD: raw mean difference.

**Table 3:** Summary of meta-regression analysis related with average daily gain (kg/d).

Covariate	Slope	*p-Value*	Intercept	*p-Value*
Initial body weight	−0.015	0.194	0.537	0.109
Experiment period	−0.005	0.459	0.714	0.380
Number of animals per group	−0.001	0.015	0.367	0.002

## Data Availability

The datasets generated and analyzed in the current study are available from the corresponding author upon reasonable request.
